# Electromigrated electrical optical antennas for transducing electrons and photons at the nanoscale

**DOI:** 10.3762/bjnano.9.187

**Published:** 2018-07-11

**Authors:** Arindam Dasgupta, Mickaël Buret, Nicolas Cazier, Marie-Maxime Mennemanteuil, Reinaldo Chacon, Kamal Hammani, Jean-Claude Weeber, Juan Arocas, Laurent Markey, Gérard Colas des Francs, Alexander Uskov, Igor Smetanin, Alexandre Bouhelier

**Affiliations:** 1 Laboratoire Interdisciplinaire Carnot de Bourgogne, CNRS-UMR 6303, Université Bourgogne Franche-Comté, 21078 Dijon, France; 2 P. N. Lebedev Physical Institute, Leninsky pr. 53, 119991 Moscow, Russia; 3 ITMO University, Kronverkskiy pr. 49, 197101 Sankt-Petersburg, Russia

**Keywords:** electromigration, Fowler–Nordheim, hot-electron emission, inelastic electron tunneling, optical antennas, transition voltage, tunnel junction

## Abstract

**Background:** Electrically controlled optical metal antennas are an emerging class of nanodevices enabling a bilateral transduction between electrons and photons. At the heart of the device is a tunnel junction that may either emit light upon injection of electrons or generate an electrical current when excited by a light wave. The current study explores a technological route for producing these functional units based upon the electromigration of metal constrictions.

**Results:** We combine multiple nanofabrication steps to realize in-plane tunneling junctions made of two gold electrodes, separated by a sub-nanometer gap acting as the feedgap of an optical antenna. We electrically characterize the transport properties of the junctions in the light of the Fowler–Nordheim representation and the Simmons model for electron tunneling. We demonstrate light emission from the feedgap upon electron injection and show examples of how this nanoscale light source can be coupled to waveguiding structures.

**Conclusion:** Electromigrated in-plane tunneling optical antennas feature interesting properties with their unique functionality enabling interfacing electrons and photons at the atomic scale and with the same device. This technology may open new routes for device-to-device communication and for interconnecting an electronic control layer to a photonic architecture.

## Introduction

The constant evolution of information technologies requires the integration and development of complex processing functionalities. The fast increasing demand of connectivity between devices necessitates the deployment of new data-transfer strategies. Optical fiber input/output pigtail-wiring connections are the key technology enabling fast and reliable data transfer down to on-card system level. However, the need for parallel processing and the physical size of these optical buses prevent their deployment as a sustainable technology for short-range on-chip interconnects. Alternative propagation supports are being developed to meet integration requirements. Silicon-based photonics for instance is offering a cost-effective strategy to merge microelectronics and photonics [1–2] and address the next generation of interchip and intrachip optical interconnects. Optical and electrical cross talk between vertical interconnect accesses, thermal envelope, footprint, wafer-bonding requirements, and the drastic increase of power consumption with the number of links are limiting factors for using this platform at the nanoscale. In parallel, the integration of alternative chip-scale routing networks is being developed [3]. For instance wireless radio-frequency (RF) data transmission between distant nodes is emerging as an alternative for wired physical waveguiding channels [4]. This approach is enabled by the availability of complementary metal-oxide semiconductor (CMOS)-compatible transceivers [5] and may offer cost-effective robust interconnects operating with a large bandwidth [6]. Yet, the implementation of hardware components for wireless network-on-chip (WNoC) is constrained by the physical size of the transmitting antennas. For instance, ultra wide-band RF interconnects and millimeter-wave traffic are relying on device sizes comparable to dimension of the chip itself (millimeters). A new paradigm is thus required to develop miniature antennas enabling future WNoC to operate with sub-micrometer transmitting units [7].

In this context, optical antennas are offering an interesting technological route to meet this integration requirement. Optical antennas are devices operating at frequencies from visible light to infrared [8]. They were primarily developed to enhance light–matter near-field interactions [9] via the excitation of surface plasmons for metal-based devices [10] or Mie resonances for dielectric antennas [11–12]. Interestingly, optical antennas have radiating properties bearing similarities with traditional RF antennas [13–16] but have a nanometer-scale footprint offering thus unsurpassed integration capability. However, the deployment of such components for interconnects has not been a viable solution so far as optical antennas are typically used as wave-vector converters to manipulate optical fields. Recent developments showed that a novel generation of optical antennas can be electrically connected [17]. These devices are operating based upon the unique properties of tunnel junctions. In particular, they can be deployed as electro-optical transceivers because they may either emit light upon injection of electrons [18–21] or optically rectify an incident electromagnetic wave [22–27]. Additionally atomic-scale gaps provide a unique test bed to identify the rules governing the physics of electron interaction with surface plasmons and photons: quantum effects were shown to challenge the classical plasmonic description [28–30] and the fluctuations of the electronic current impart a rich photon statistics [31–32].

In this work, we present a strategy to realize electrically connected optical antennas by employing the electromigration of metal nano-constrictions. The atomic-scale gap acts as an active feedgap operating a transduction between an electrical signal and an optical radiation. We electrically characterize the device and deduce the relevant properties using the standard description of tunneling transport. We demonstrate light generation from the feedgap and discuss different emission mechanisms based on the radiated spectrum and activation voltage.

## Results and Discussion

### Nanofabrication of electrically connected optical antennas

**Figure 1:**
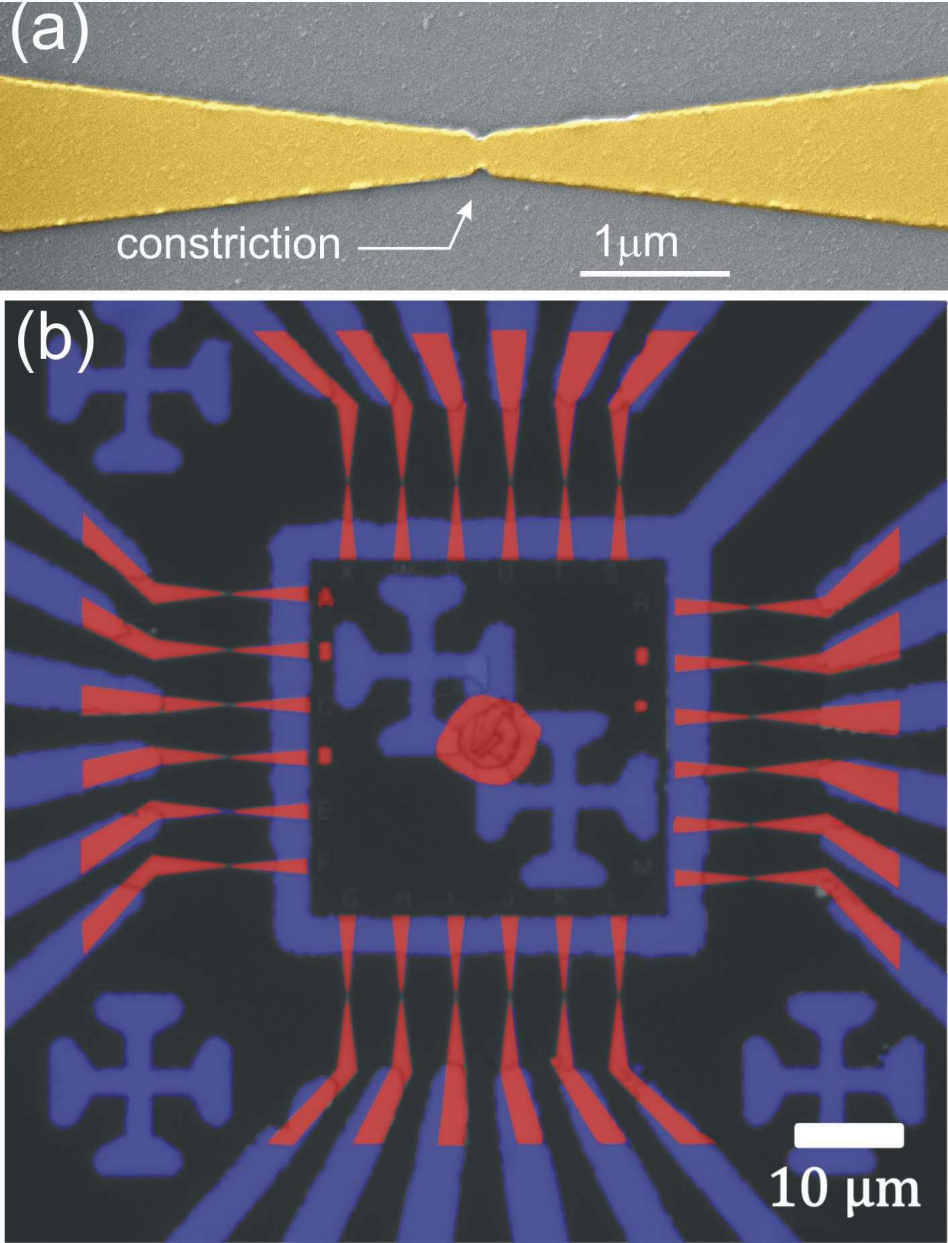
(a) False-color scanning electron micrograph of a typical constriction separating two tapered electrodes. The yellow color indicates the Au part. The constriction is 150 nm wide for a length of 70 nm. (b) False-color image of a series of constrictions and their electrical connections. The areas colored in red are made by electron-beam lithography, the regions in blue are those fabricated by photolithography.

The starting geometry for realizing an electrically connected optical antenna is a Au constriction formed between two fan-out electrodes laying on a glass cover slip. We use electron-beam lithography and standard physical vapor deposition to produce gold constrictions and the proximity electrodes. The thickness of the Au layer is typically 50 nm, and we use a 2 nm thick Cr or Ti adhesion layer to improve gold adhesion on the glass substrate. A scanning electron micrograph of a pristine 150 nm wide Au constriction formed between two bow-tie leads is exemplified in Figure 1a. The electrical connections of the constriction to outside control electronics is obtained in a second step of fabrication by ultraviolet (UV) photolithography. An image of a typical sample, constituted of 24 constrictions and their associated macroscopic electrodes, is illustrated in Figure 1b. The red and blue regions are realized by electron-beam lithography and UV lithography, respectively. Each electrode is connected to a common ground (centered square) and can be individually addressed by a set of peripheral electrodes.

To create a tunnel junction that will eventually form the active feedgap of an optical antenna, we perform an operator-controlled electromigration of the constriction. Electromigration is, in a broad sense, the transport of mass due to an electric current passing through a metal. The phenomenon depends on several variables, such as current density, temperature, composition, stresses in the solid, and grain structure [33]. We adapted and tested different strategies available from the literature ranging from the simple ramping of an applied voltage until breakdown to approaches relying on feedback mechanisms [34]. We finally settled on a method where the applied bias is manually adjusted to control the time evolution of the conductance of the constriction. The procedure is as follows: An ac voltage applied across the constriction with an amplitude *V*_ac_ = 20 mV and a frequency *F* = 12.6 kHz is summed to an adjustable direct current (dc) bias *V*_dc_. *F* is used as an external reference for a lock-in amplifier. A current-to-voltage amplifier converts the current flowing through the device to a voltage output read by the lock-in amplifier. The output of the lock-in is proportional to the amplitude of the modulated current oscillating at *F*. The conductance of the constriction *G* is then estimated by dividing the lock-in signal by *V*_ac_. The conductance of the devices before electromigration is in the range of 1 to 4 mS, and includes the contribution from leads and contacts. *V*_dc_ is then incremented by steps of 100 mV. We monitor *G*(*t*) during each step and the entire electromigration process may be divided into phases.

**Figure 2:**
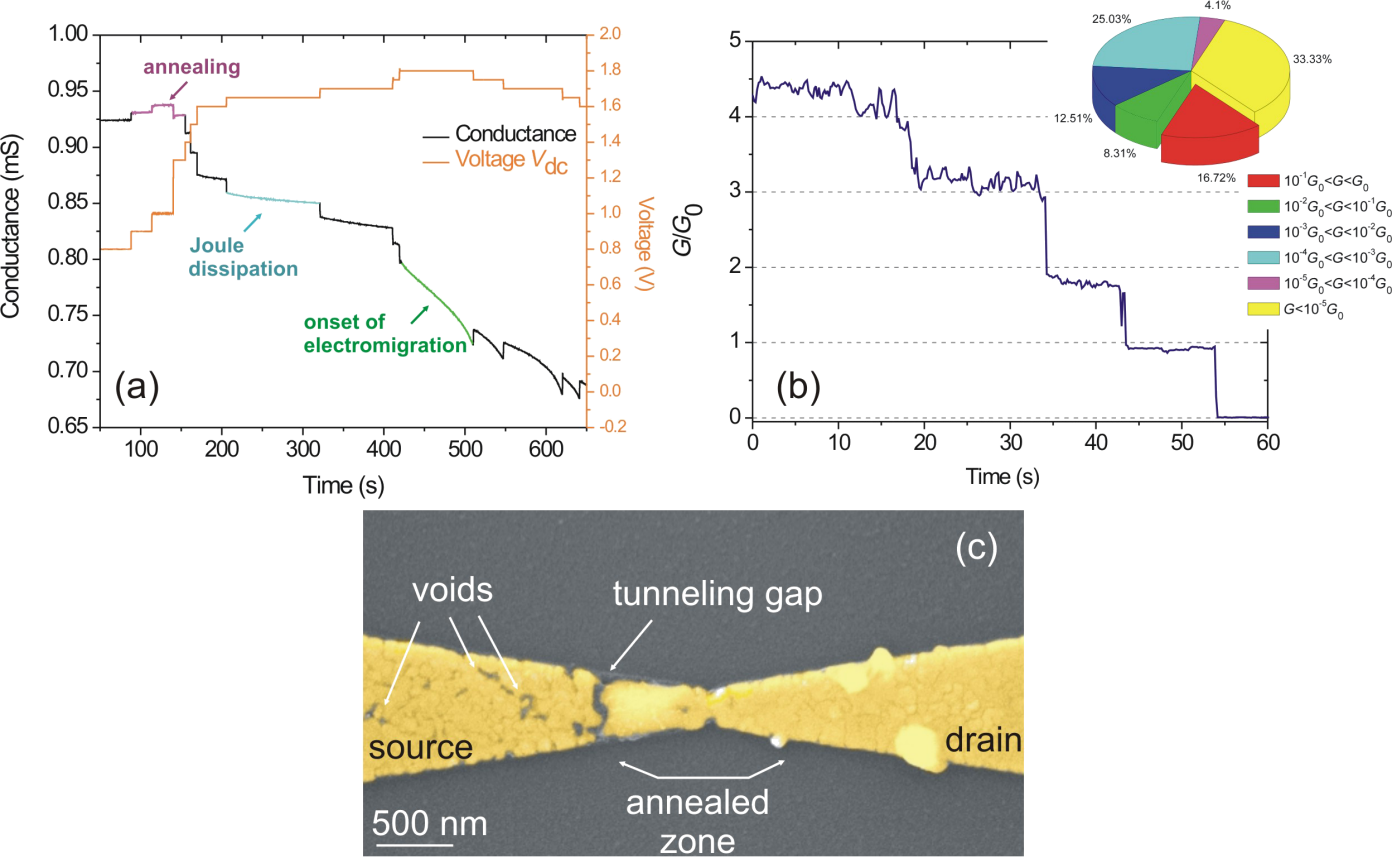
(a) Temporal extract of the electromigration sequence featuring the effect of partial annealing, Joule heating and onset of electromigration on the evolution of the condutance with bias increments. (b) Time trace of the conductance *G*(*t*) during the last moments of the electromigration process. The conductance is quantized in units of *G*_0_, the quantum of conductance. Inset: statistics on the final conductance for a series of 24 electromigrated constrictions. (c) False-color scanning electron micrograph of the device after electromigration. The tunneling junction forms on the source electrode. The zone corresponding to the constriction has been annealed by Joule dissipation during the process, reducing the number of grain boundaries available to trigger electromigration. Voids resulting from Au migration are also observed on the source electrode. The image is obtained by sputtering a thin conductive Au layer on the post mortem device.

During the first few *V*_dc_ steps, *G*(*t*) is fairly constant because Joule dissipation is not yet affecting the temperature-dependent resistivity of gold. On increasing *V*_dc_, the temperature of the constriction grows and the conductance starts to fluctuate. The general trend is that *G*(*t*) decreases when stepping up *V*_dc_. We also consistently observe a momentary rise of the conductance, which we attribute to the desorption of surface contaminants as well as a temperature annealing of the constriction due to dissipation of the electrical power in this area. This effect can be traced in Figure 2a at around *t* = 150 s. An example of another temperature effect is illustrated at *t* = 200 s in Figure 2a. The applied bias is constant, but *G*(*t*) decays towards a stable value. This is understood from the temperature-dependent resistivity of the material: For a given *V*_dc_ the current flowing in the constriction dissipates heat and affects in return the temperature-dependent resistivity [35]. These conductance fluctuations are typically observed for *V*_dc_ ≤ 1.7 V and corresponds to the end of the first phase of voltage increments.

When applying higher voltages, *G*(*t*) generally drops with a rate rapidly increasing with time. The process is entering a second phase. This behavior is the signature of the onset of electromigration of the device, and will rapidly lead to the failure of the constriction if the applied bias is maintained. In order to control the electromigration process, we reduce *V*_dc_ by 100 mV when the dropping rate of *G*(*t*) exceeds 5 μS·s^−1^. This usually stops the runaway momentarily. An example of the procedure is illustrated at *t* = 420 s in Figure 2a. After a few seconds, the conductance drop resumes and *V*_dc_ is again adjusted to control the decrease of the conductance. When *G*(*t*) stays constant, the electromigration is hindered because the temperature of the constriction is too low to thermally assist the process [36]. *V*_dc_ is consequently increased by a few increments to trigger the process again. The constriction will eventually break for bias voltages *V*_dc_ of around 500 mV. We sometimes observe quantized conductance steps indicating the change of transport regime from diffusive to ballistic as illustrated in Figure 2b. The conductance is normalized by the quantum of conductance *G*_0_ = 2*e*^2^/*h* = 77 μS, where *e* is the electron charge and *h* is Planck’s constant. The passage to the tunneling regime when *G < G*_0_ ends the electromigration process. In the inset of Figure 2b, we show a statistics of the final conductance values measured after the electromigration of a series of 24 constrictions. About 17% of the devices feature large conductances approaching *G*_0_.

A scanning electron image (SEM) of an electromigrated constriction is displayed in Figure 2c. Electron imaging is made possible by sputtering a thin conductive Au layer. Different information can be deduced from the image. To begin with, the tunneling gap is not situated at the location of the constriction, but is displaced towards the source electrode as already reported in the past [37]. There are two reasons why the formation of the gap is not occurring at the constriction. First, the dissipation of electrical power during the process takes place at the constriction, i.e., at the region of highest resistance [38]. The evaporated Au layer can thus be partially annealed through Joule heating. We substantiate this hypothesis by the temporary improvement of the conductance at *t* = 150 s before the onset of electromigration discussed in Figure 2a. The SEM image of Figure 2c is also providing additional confirmation of a partial annealing of the constriction. In the area marked “annealed zone” in the image, the concentration of triple points considerably reduces and the dimensions of grain sizes increase. The constriction is thus less susceptible to failure because diffusion of atoms is facilitated by these polycristalline structural defects [39]. Secondly, the gap is occurring at the source electrode because charge carriers here first collide with grain boundaries to initiate atom diffusion as it can be seen by number of voids present in this electrode (Figure 2c).

### Electrical characterization

**Figure 3:**
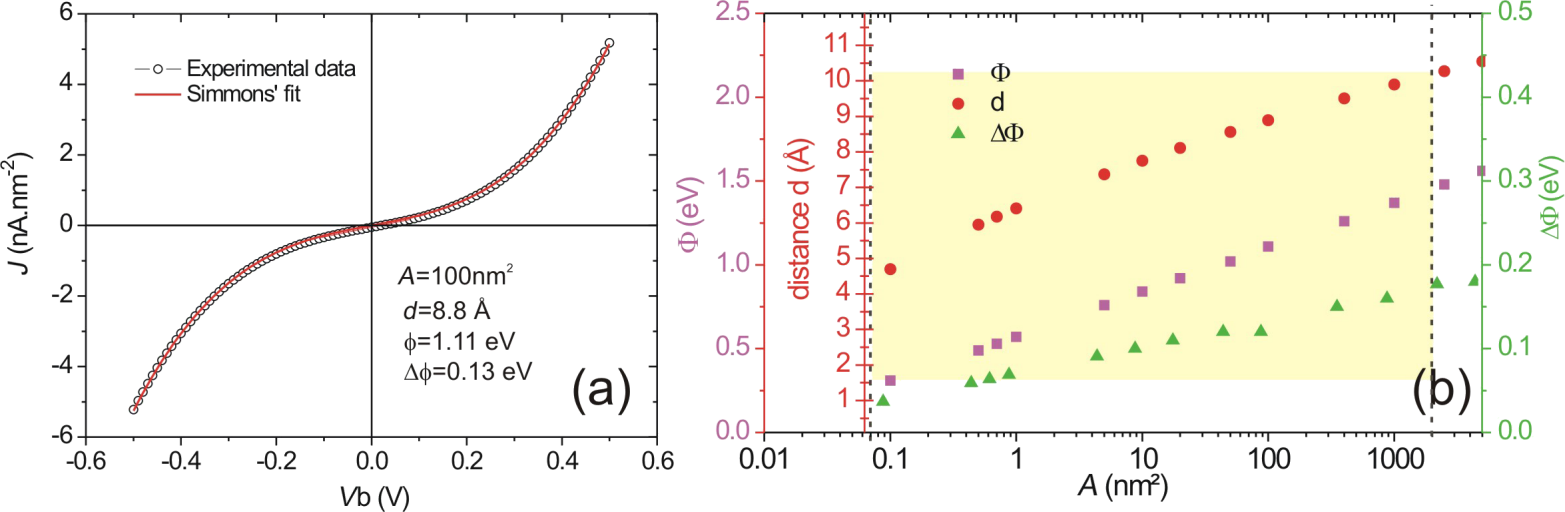
(a) Current density *J*_T_ plotted versus applied bias *V*_dc_. The black circles are experimental data points and the solid red line is the best fit to the data using Simmons’ model of tunneling transport. (b) The fitting parameters 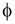, Δ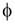, and *d* as functions of the junction area *A*. The yellow frame represents a parameter space consistent with respect to the experimental measurements.

The electrical characterization of the tunneling feedgap forming the active area of the optical antenna primarily consists of measuring the current-to-voltage characteristics *I*_T_(*V*_dc_). An example is illustrated in Figure 3a. In this graph, the current density (*J*_T_ = *I*_T_/*A*) is displayed for an arbitrary tunneling junction area *A*, chosen at 100 nm^2^.

For a tunneling barrier subject to a small applied bias, the transport may be described by Simmons’ equation of tunneling electrons [40]:

[1]
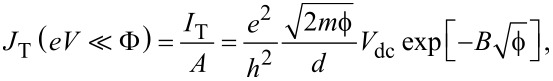


where 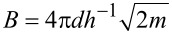, *A* and *d* are the effective area and width of the junction, *m* is the electron mass, and *h* is Planck’s constant. The average barrier height 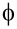 includes the presence of an image potential that reduces the barrier height. Considering that the work function of gold is ca. 5.4 eV, Equation 1 is usually valid for applied bias voltages up to a few volts.

The shape of the tunneling characteristics of *I*_T_(*V*_dc_) essentially depends on the parameters *d* and 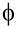 with minor corrections from the effective area *A* [41]. These parameters can be extracted by fitting the experimental characteristics. Generally, the current-to-voltage plots measured from electromigrated gaps are not symmetrical with respect to the applied bias. The irregularities of the gap, such as protrusions, affect the two sides of the energy barrier, 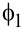 and 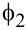, differently [42] leading to asymmetric output characteristics. Electron tunneling through an asymmetrical trapezoidal barrier is described following Brinkman’s formalism [43]:

[2]
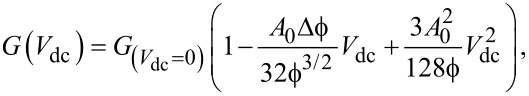


with 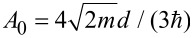 and 
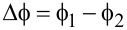
. 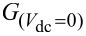 is the zero-bias conductance:

[3]
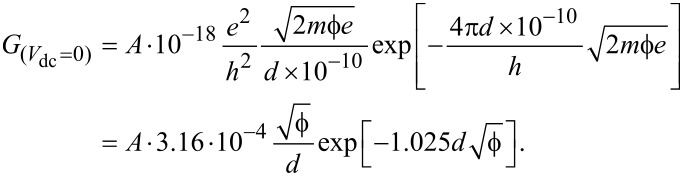


*d* is here in angstroms, *A* is in square nanometers, *e* is in coulombs and 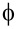 is in electronvolts. Combining Equation 2 and Equation 3, the current density *J*_T_(*V*_dc_) is

[4]
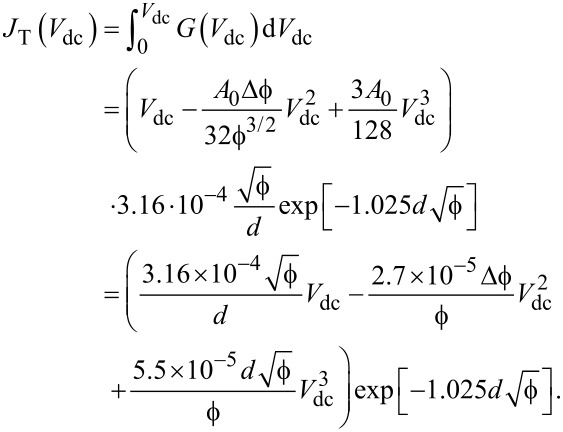


The set of parameters [*d*, 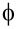 and Δ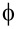] is determined by conducting a fit to the experimental data as shown by the red curve in Figure 3a. We arbitrarily set the effective area to *A* = 100 nm^2^ because we cannot obtain a precise experimental determination of the active area in which the electrons tunnel from the complex morphology of electromigrated gaps. In Figure 3b, we explore the dependence of *d*, 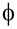 and Δ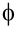 on the area *A*. The yellow region in the graph shows the boundaries of the parameter space, limited on one side by half of the area occupied by a gold atom, and on the other side, by the cross-sectional area of the constriction. Even with a variation of *A* by four orders of magnitude, the estimated gap size remains at *d <* 1 nm. Such a small distance between two electrodes is the key characteristics for developing the novel generation of electro-optical antennas discussed here.

It is interesting to display the characteristics of *J*_T_(*V*_dc_) using the so-called Fowler–Nordheim representation to understand the physical meaning of the average barrier height 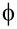, which is in the region considered in Figure 3b considerably lower than the work function of gold. The Fowler–Nordheim plot, 
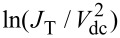
 as a function of 1/*V*_dc_, is commonly used to distinguish the transition between direct tunneling at low *V*_dc_ and a high-bias regime where the energy barrier is drastically reduced and electrons are tunneling by field emission [44]. Thus, the representation isolates two extreme cases of bias polarization: 
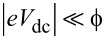
 and 
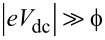
 separated by a minimum in the plot indicating the cross-over between the transport regimes. The effective barrier height of the electrodes may be directly inferred from this minimum, commonly referred to as the transition voltage *V*_t_.

**Figure 4:**
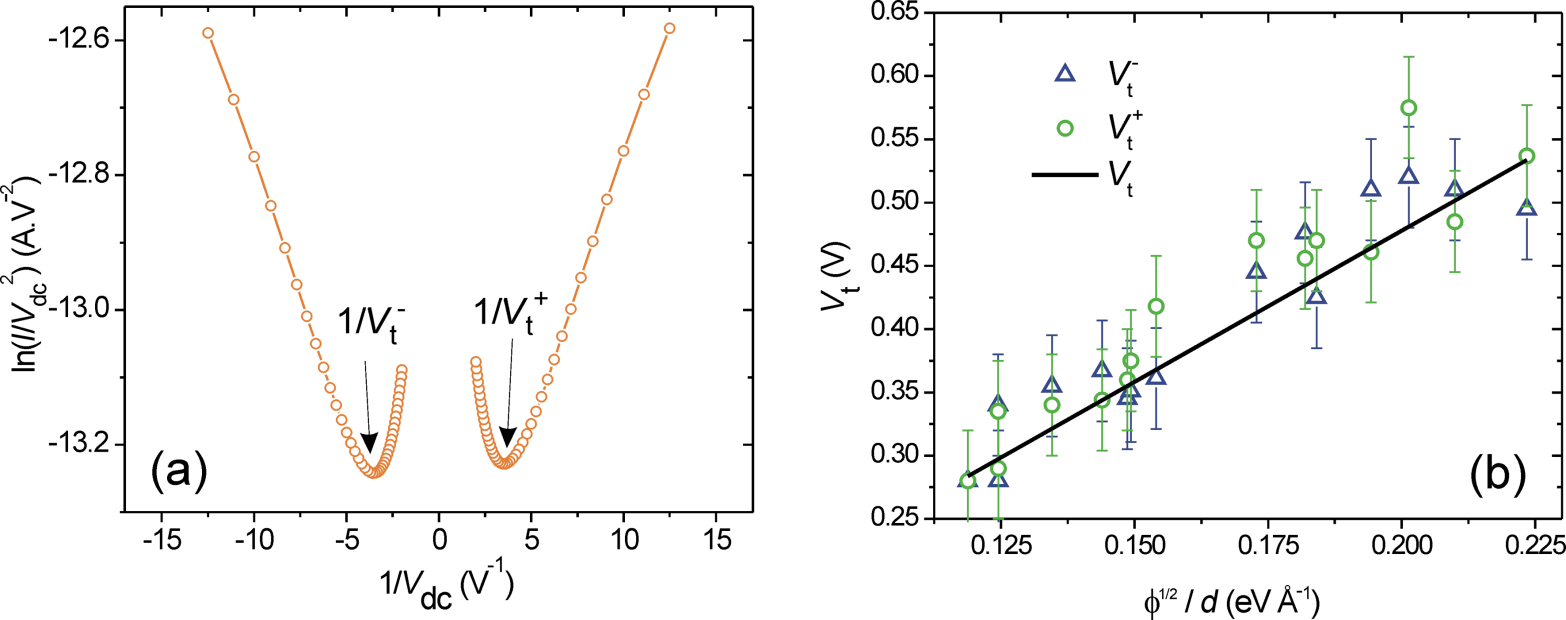
(a) Fowler–Nordheim representation of the *J*_T_(*V*_dc_) data shown in Figure 3a. The transition voltages 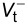 and 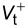 are determined from the inflection points of the representation. (b) 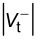 and 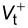 as functions of 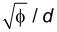 for 15 electromigrated junctions. The solid line is the transition voltage *V*_t_ calculated with Equation 5 and the parameters 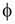 and *d* deduced from Simmons’ fit of the electrical characteristics.

The Fowler–Nordheim plot of the *J*_T_(*V*_dc_) characteristics discussed in Figure 3 is displayed in Figure 4a. Two clear minima are identified with respect to bias polarity at nearly symmetric values 1/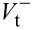 = −3.51 ± 0.16 V^−1^ and 1/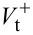 = 3.5 ± 0.09 V^−1^, corresponding to an average transition voltage |*V*_t_| = 0.28 V. It is immediately obvious here that the transition voltage inferred from the Fowler–Nordheim plot differs significantly from the average barrier height resulting from Simmons’ model (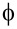 = 1.11 eV). However, such a low value of 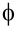 is consistently reported in Au tunnel junctions [19,26,41,45] and is tentatively attributed to surface states on the electrodes [41,46], the effect of image charges [47], the presence of protruding atoms [48] or a Schottky contact [45]. Despite this body of work, the interpretation of the Fowler–Nordheim plot and the transition voltage has been debated in the past. Huisman et al. [49] followed by Vilan et al. [50] argued that the inflection in the Fowler–Nordheim plot is a generic property of the nonlinear characteristics and takes place when the third-order term in Equation 2 becomes important. By recasting Equation 2 in the Fowler–Nordheim form, we find an analytical expression for *V*_t_ by searching the minimum of the function:

[5]
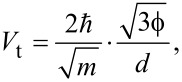


where 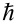 is in electronvolts and *d* is in angstroms. Equation 5 shows that the transition voltage does not depend on the barrier height 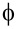 but on the ratio between the square root of the average barrier height and the barrier width, 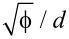. Huisman derived a similar expression [49] using Stratton’s tunneling formalism with a dependence on 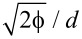. Inserting the values of 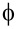 and *d* deduced from Simmons’ fit to Equation 5 leads to *V*_t_ = 0.28 V, which is exactly the value inferred from the Fowler–Nordheim plot. To reinforce this interpretation of the transition voltage, we plot in Figure 4b the 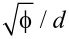-dependence of the transition voltages measured on either side of the Fowler–Nordheims plot for a series of 15 electromigrated junctions. The solid black curve is |*V*_t_| calculated using Equation 5 and the parameters 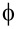 and *d* extracted from fitting the experimental data of *J*_T_(*V*_dc_) with Equation 4. The dependence of the transition voltage on 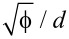 is clearly revealed from the graph, demonstrating that *V*_t_ cannot be an estimate of the tunneling barrier height.

Even if the Fowler–Nordheim plot of the device shown in Figure 4a feature a symmetric transition voltage with respect to the bias polarities, electromigrated junctions may have asymmetric current-to-voltage characteristics; the data points representing 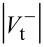 and 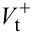 in Figure 4b do not generally coincide. This is expected from the irregular morphology of the junction and its influence onto the barrier height [51].

### Light-emitting electron-fed optical antennas

The electromigrated planar junctions discussed above may serve as light-emitting optical antennas when electrons are injected in the tunnel barrier formed between the two metal leads. The junction acts as the antenna feed because radiation is emitted from this driven element [52].

**Figure 5:**
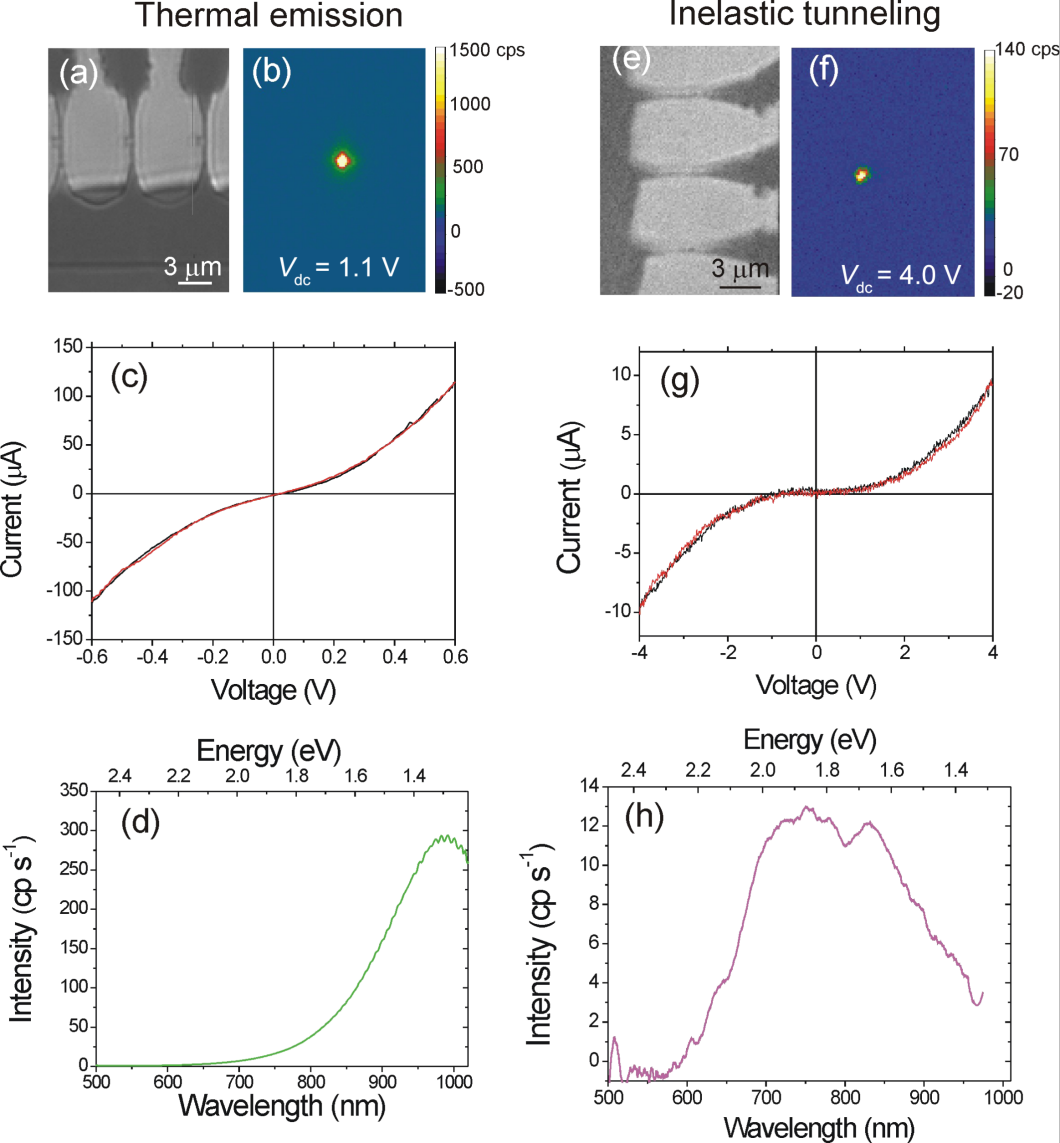
(a) Transmission optical image showing a series of electromigrated constrictions. (b) Optical image of the light emission when the centered junction is biased at 1.1 V. (c) Corresponding current-to-voltage characteristics where the black and red curves are the forward and backward voltage sweep, respectively. (d) Emission spectrum recorded for *V*_dc_ = 0.9 V. The entire spectrum violates the quantum cutoff since *h*ν *> eV*_dc_. (e) and (f) are images of another series of electromigrated gaps showing the respective layout of the structure and the optical activity when the center junction is biased at 4.0 V. (g) Corresponding current-to-voltage characteristics. (h) Electroluminescent spectrum of the light-emitting device obtained at *V*_dc_ = 4 V. The emission is characteristic of inelastic electron tunneling events with *h*ν *< eV*_dc_. The spectra are corrected for the quantum efficiency of the CCD camera and the transmission of the microscope.

Light emitted from tunnel junctions has been heavily investigated since the pionner work of Lambe and McCarthy [53] and is generally attributed to inelastic scattering of tunneling electrons into radiative surface plasmon modes. Generally, spectra detected from electrically-driven on-chip optical antennas are in agreement with this picture [19–21,27,54]. However, some authors reported an over-bias emission from atomic contacts where the emitted spectra are no longer limited by the kinetic energy of the electrons given by the applied bias [18,55–56]. In these devices, the emission is described by thermal radiation of an out-of-equilibrium heated electron distribution, which is promoted by the electron–electron interaction dynamics. Both light-emission mechanisms may be observed in the electromigrated junctions discussed in the previous section as illustrated in Figure 5. Prevalence of inelastic scattering in the barrier over electronic heating essentially depends on the conductance of the device and the current density flowing through the contact. Typically, the quantum cutoff *h*ν *< eV*_dc_ is violated when 0.1*G*_0_ *< G < G*_0_, where ν is the frequency of the photon.

In the left column of Figure 5, we show an example of emission from a hot distribution of carriers for an electromigrated junction biased at *V*_dc_ = 1.1 V. The light generated by the tunnel junction upon electrical biasing and emitting in the substrate is collected using an inverted optical microscope (Nikon, Eclipse) equipped with a 100×, 1.49 numerical aperture (N.A.) objective and a charge-coupled device (CCD) camera recording the object plane of the microscope (Andor, Luca EM S 658M). The resolution of the microscope is about 300 nm at 800 nm emission wavelength. The optical activity in Figure 5b is restricted to the location of the gap (not distinguishable in the optical transmission image of Figure 5a). The electrical characteristics (Figure 5c) feature a value of 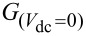 = 69 μS = 0.9*G*_0_. Figure 5d shows the emission spectrum of the device taken at *V*_dc_ = 900 mV. In the framework of inelastic electron tunneling, no light should be detected in the sensitivity window of Si-based devices because at *V*_dc_ = 900 mV, the wavelength at the quantum cutoff is 1300 nm to which the Si detectors are blind. Clearly, the spectrum is violating this quantum cutoff. Here, this overbias response is understood from the spontaneous emission of hot carriers, accelerated by the electric field present at the junction, and colliding with the boundary of the gap [18]. The detected spectrum is thus the visible tail of a thermal peak located in the infrared. The position of peak is not directly related to the bias via the quantum relation cited above, but to the electronic temperature of the hot electrons responsible for the emission. In our previous report on analogous devices [18], electron temperatures exceeding 1000 K were measured for similar operating conditions, which pushes the thermal peak roughly between 2 and 3 μm.

The column on the right of Figure 5 illustrates an example of electromigrated junction operating in a different emission regime. Here the device is biased at *V*_dc_ = 4.0 V. Very much like the thermal glow of Figure 5b, the active region is restricted to the tunnel gap, but the electrical characteristic (Figure 5g) gives 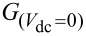 = 5.6·10^−8^ μS = 7·10^−4 ^*G*_0_. The spectrum emitted by the optical tunneling gap antenna is consistent with the quantum cutoff; the energy of the detected photons is smaller than the kinetic energy of the tunnel electrons. The substantial drop of intensity below 600 nm is due to the onset of interband transitions in the material. For this device, the emission is probably resulting from the radiative decay of surface plasmons populated by inelastic tunnel electrons [19–21].

In the following section we show examples of devices where light emission from tunneling electron-fed optical antennas is not simply radiated in free-space but partially coupled to waveguiding architectures. Here, the objective is to implement a first technological step to integrate compact transducing tunnel optical antennas directly at the input port of photonic links to provide for an electronically driven optical transmission line with broadband spectral characteristics. This asset may help at increasing the bandwidth via wavelength-division multiplexing. Integrated broadband sources are also utilized in photonic sensing chips [57] or to command non-classical secondary photon sources [58]. There is thus a demand for versatile and low-cost integrated light sources, and optical tunneling gap antennas may provide an alternative technology to solid-state light emitting diodes or quantum dots. Coupling of such a junction have been recently demonstrated in plasmonic strips [27,59] and we extend the concept to dielectric TiO_2_ waveguides and slot geometry.

#### Electrically connected optical antennas on TiO_2_ waveguides

Because the emission spans the visible part of the spectrum, and, depending on the underlying mechanism, extends to near-infrared wavelengths, standard silicon-based platforms are not adapted to collect and guide photons emitted by the junctions. For the operation at visible wavelengths waveguiding structures composed of TiO_2_ feature interesting material properties [60–61] such as broadband transparency, high refractive index, compatibility with complementary metal-oxide semiconductors and ease of processing.

**Figure 6:**
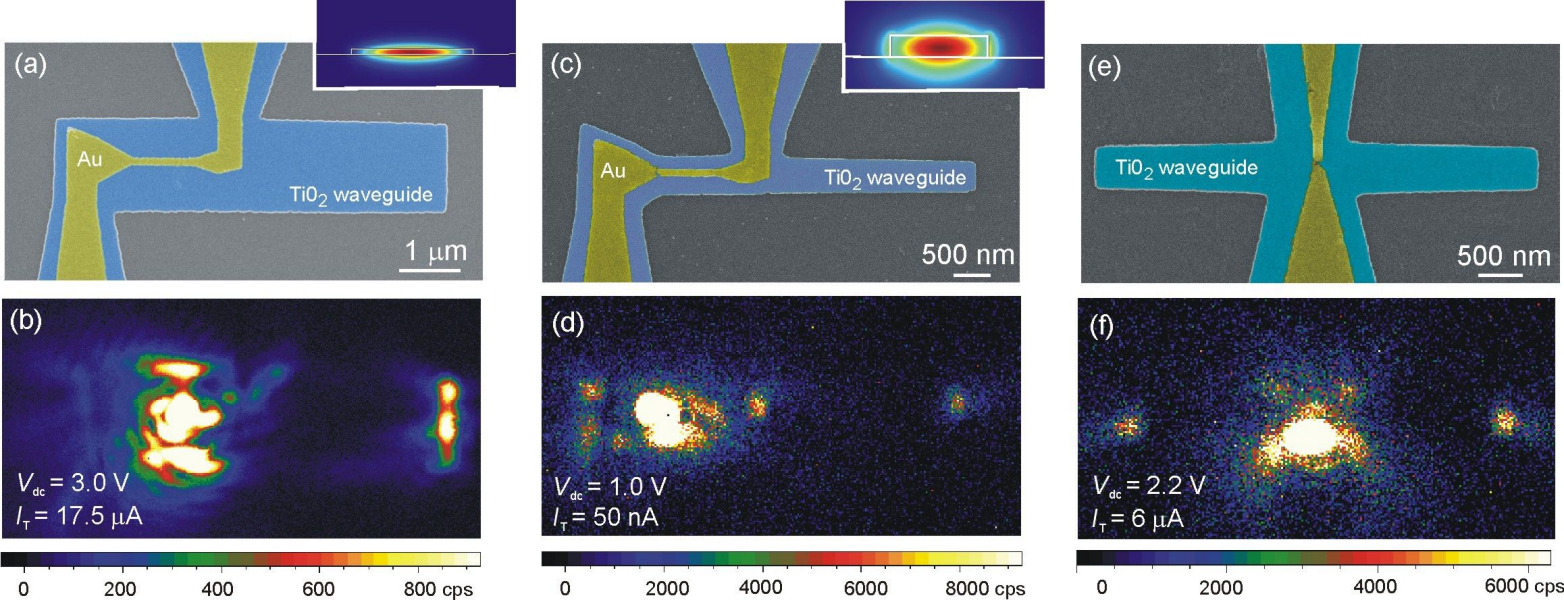
(a, c, e) Colorized scanning electron micrographs of the electron-fed optical antennas integrated in various waveguiding geometries. The yellow and blue hues show the Au and TiO_2_ materials, respectively, and the glass substrate appears in gray. The TiO_2_ waveguides are 1.5 μm wide in (a) and 500 nm wide in (c) and (e). The Au electrodes are parallel to the waveguide axis in (a) and (c) while in (e), the orientation of the electrodes is perpendicular to the two symmetrical TiO_2_ stripes. Insets: Calculated modal distribution of the electric field norm in the waveguides evaluated at 800 nm. (b, d, f) False-color CCD images of the distribution of light in the structure upon electrical biasing of the tunnel junction. The electrical conditions are given in the images and the integration time is 30 s for all frames. The color scale is saturated to enhance the contrast.

We realize the implementation of tunneling antennas on a TiO_2_ waveguide through a multi-step process. First, a 85 to 110 nm thick titanium dioxide layer is deposited by physical vapor deposition on a clean glass substrate. Then, the Au backbone that will subsequently define the electrically connected tunneling optical antennas is fabricated by electron-beam lithography, Au evaporation and lift-off process. The structures consist of either a gold nanowire of 1.1 μm length and 130 nm width or a constriction as discussed above. For both types, the structures are connected to a set of electrodes. The thickness of the nanowire and electrodes is 50 nm, including a 5 nm Ti adhesion layer. The third step is the dry etching of the TiO_2_ layer. For that, we first create an etching mask by electron-beam lithography, thermal deposition of a 30 nm thick nickel layer and lift-off. Reactive ion etching is then used to remove the TiO_2_ layer and to define the waveguides. More details about this etching process can be found in [62]. A last optical lithography step is carried out to define the macroscopic leads that are connected to the microscopic electrodes. The process is subsequently terminated by electromigrating in situ the nanowire to create the optical tunneling gap antenna. Figure 6a,c,e illustrates the experiment with different waveguide geometries and gap orientations. In Figure 6a, the TiO_2_ waveguide is 85 nm thick and 1.5 μm wide, and the SEM image was taken before the electromigration of the nanowire. In Figure 6c and Figure 6e the waveguides have a cross section of 500 nm × 110 nm and both images were taken after creating the optical tunneling gap antennas. Note that in Figure 6e, the displacement of the junction towards the source electrode has been taken into account to place the tunneling gap at the center of the waveguide.

Figure 6b,d,f shows false-color CCD images showing the distribution of the light radiated in the substrate when the tunneling junctions are biased by *V*_dc_. The electrical operating conditions are reported in the images. In all these devices, light is most likely emitted by inelastic tunneling because the detected spectral range is below the bias energy: *h*ν *< eV*_dc_. The zero-bias conductance values are all about 10^−2^*G*_0_.

In the set of images in Figure 6, the strongest signal detected through the glass slide originates from the junction itself. However, the images show also that light emerges from the distal end of the TiO_2_ stripes indicating that a portion of the power emitted by the electron-fed antennas is coupled to the dielectric structure and is transmitted away from the radiating feedgap. The optical tunneling gap antenna may therefore be used as an electrically activated local source of light. An absolute coupling efficiency is difficult to estimate since only the light emitted in the substrate is collected here. Nonetheless, we may qualitatively evaluate an effective coupling yield and compare devices. We numerically assess the characteristics of the mode supported by the TiO_2_ waveguides by a two-dimensional finite element calculation (Comsol software) using published values of the refractive index of TiO_2_[63]. The insets of Figure 6a and Figure 6c display cross-sectional views of the norm of the electric field existing in the waveguides at a wavelength of 800 nm. At the operating voltage, the emission of the electron-fed antenna is typically spanning the visible and near-infrared spectral region. For the TiO_2_ geometries discussed here, the confinement loss is calculated to be about 10^−2^ dB for a 10 μm long waveguide. We may therefore neglect propagation losses in the TiO_2_ while estimating the coupling ratio. In Figure 6b, the light collected at the waveguide termination is about 12% of the total signal received on the detector. Optimizing the amount of power emitted by the local source and transferred to the waveguided modes requires to shape the wavevector distribution radiated by the antenna and to reduce the influence of the electrode. This may be achieved by introducing multi-element antennas [16,64]. The electromigrated gap features the characteristic of a dipolar source [18] despite the marked nanometer-scale irregularities of the gap itself. Structuring the immediate environment of the junction, i.e., the feed of the antenna, with elements acting as a reflector and directors may help in shaping the broadband-emission diagram emitted in the dielectric and improve the overlap with photonic modes. There are also reports of efficient coupling by engineering a cladding material surrounding the active emitting area [65], by a heterogeneous integration of the source in a structured waveguide [66–67], or by using extreme modal confinement [68].

We have tried simple steps to increase the apparent coupling yield to the modes sustained by the geometries without relying on directivity-enhanced designs or more complex engineering. As it can be observed in Figure 6b and Figure 6d, scattering takes place at the edges of the waveguide right above and below the location of the junction as well as on the Au electrodes. Thus, losses imparted by the electrodes at the top of the waveguide are present and contribute to attenuating the mode in this part of the waveguide. These scattering information further suggest that the antenna radiates also in a direction perpendicular to the main axis of the waveguide. To decrease these scattering points, we introduce the geometry of Figure 6e, where the waveguides are oriented perpendicular to the electrodes. While the amount of signal emerging from the distal ends remains modest (8% on the left port and 6% on the right port), scattering and attenuation by the electrical circuit is reduced.

#### Electrically connected optical antennas emitting in slot waveguides

**Figure 7:**
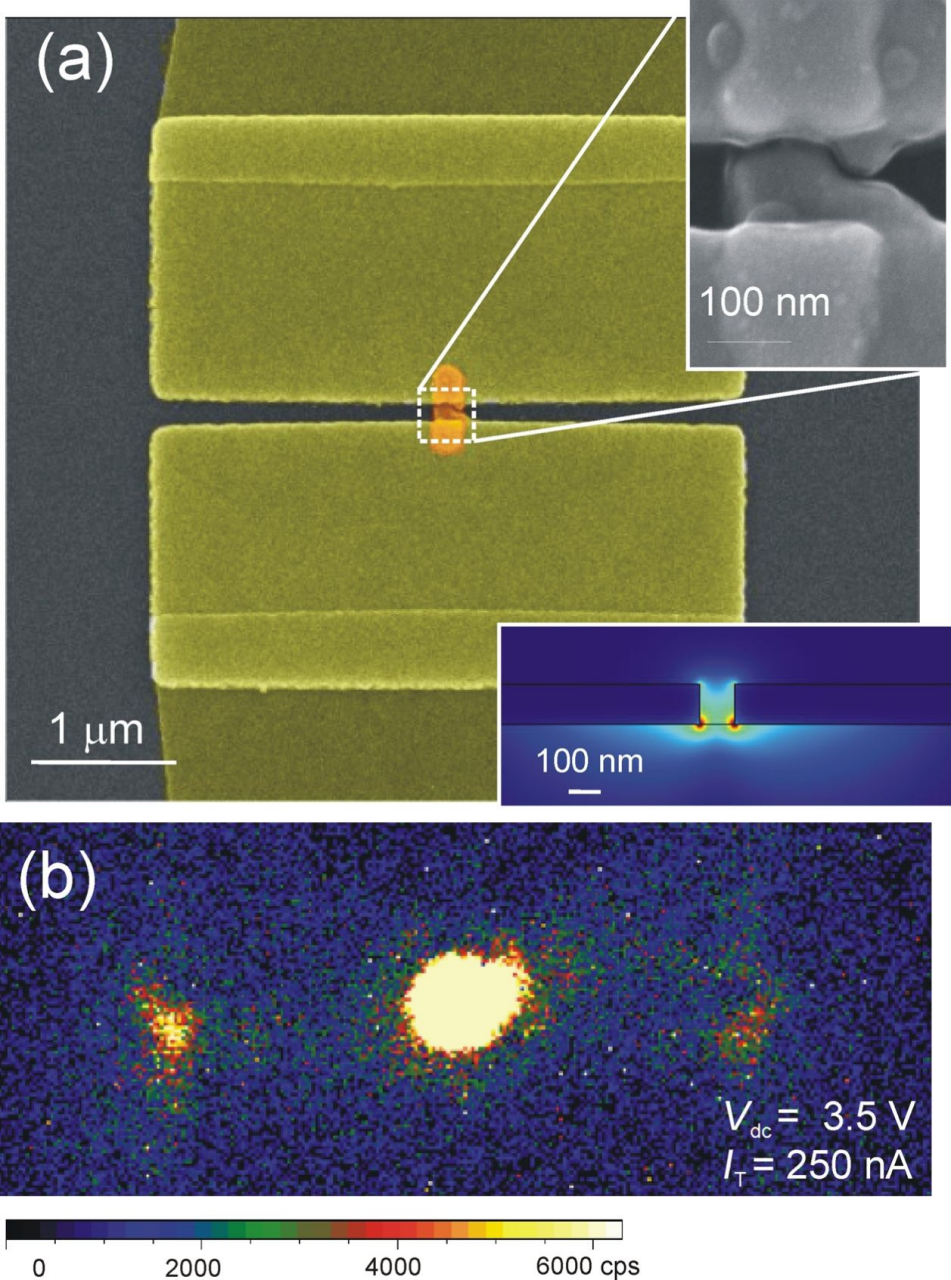
(a) Colorized image of the optical tunneling gap antenna (orange) integrated inside a slot waveguide formed by two Au pads separated by 130 nm. Upper inset: close-up SEM image of the junction after electromigration. Lower inset: calculated distribution of the norm of the electric field in the 130 nm × 150 nm slot waveguide. (b) False-color CCD image of the distribution of light when the antenna is biased at *V*_dc_ = 3.5 V.

Figure 7 shows another example of the integration of optical tunneling gap antennas. In this configuration, the electromigrated junction is located between two 130 nm thick large metallic pads forming a slot waveguide. Slot waveguides are characterized by deep modal confinement while maintaining micrometer-range propagation [69]. Excitation of the mode from free-space radiation is usually insured by the mediation of passive antenna couplers [70]. In the example shown in Figure 7, the 130 nm wide slot is directly excited in situ by the emission of the electron-fed antenna, providing thereby a simultaneous excitation and coupling strategy of the modal landscape with a self-content ultracompact device. As in the case of the TiO_2_ waveguide, the strongest detected signal originates from the junction itself. However, light is also observed at the two outputs of the slot demonstrating that confined modes can be electrically excited by the junction.

The percentage of light scattered at the slot termination represents about 10% of the total collected signal. If we take into account the attenuation of the mode, the percentage effectively coupled to the mode at the location of the source is likely to be higher. We also estimated the propagation length of the slot mode using finite-element simulations. A cross-sectional view of the norm of the electric field is shown in the inset of Figure 7 for a mode existing at 800 nm. The field is mostly located in the slot and the calculated propagation length is *L*_spp_ = 6.2 μm. If we take this attenuation into account, the percentage of the light coupled to the waveguide can be evaluated in the following manner. Starting from the intensities measured at both ends of the slot waveguide 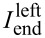 and 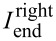, we first calculate the intensity coupled to the waveguide mode *I*_mod_ at the location of the feed:


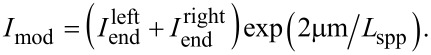


We then calculate the coupling coefficient Γ by normalizing *I*_mod_ with the total intensity collected by the CCD camera including the intensity effectively recorded at the position of the antenna *I*_feed_ and the estimated intensity delivered to the waveguided mode *I*_mod_:

[6]
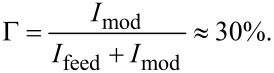


The somewhat larger coupling ratio of the antenna emission to the slot mode can be understood from the increased modal confinement compared to dielectric TiO_2_ waveguides. Because the emission is radiated from the nanometer-scale feedgap of the electromigrated junction, the emitted wavevector distribution matches better the momentum of the mode confined between the two metal electrodes.

## Conclusion

We argue in this work the potential of electromigrated in-plane tunnel junctions to act as electron-fed light-emitting optical antennas. Starting from an electrically connected gold constriction, we describe the electromigration process by a constant monitoring of the electrical conductance. We analyze the different signatures leading to the formation of the gap including the occurrence of quantized conductance steps characteristic of a ballistic transport of electrons. We detail the balanced contribution of the thermal dissipation, which is on one hand required to assist the electromigration but is, on the other hand, preventing the formation of the gap at the constriction. The electrical nonlinear properties of the tunnel junction are investigated with a standard model of electron tunneling enabling to extract crucial parameters such as the gap size and the effective barrier height. We discuss the interpretation of the Fowler–Nordheim representation and show that the transition voltage deduced from this representation not only depends on the energy barrier height as commonly reported, but also on the barrier width. We show that upon injection of electrons, the electromigrated tunnel junctions are emitting light. The emission mechanism depends on the zero-bias conductance *G* of the tunnel junction. Roughly speaking, light is generated by inelastic tunneling events when *G* is much smaller than the quantum of conductance *G*_0_ in accordance with the standard description of electromagnetic radiation produced in metal–insulator–metal devices. However, when the gap is small (few angstroms) and *G* approaches *G*_0_, the energy of the photons exceeds the energy of the electrons provided by the bias, and the emission originates from the glowing radiation of a hot electron gas. We finally demonstrate that these electron-fed optical antennas produced by electromigration can be integrated into more complex device architectures. In particular, we show that the emission released by the feedgap can be coupled to propagating modes with the efficiency approaching 30% for slot waveguides. Additional efforts are required to optimize the coupling yield, notably by developing strategies to shape the momentum and the energy of emitted photons. The devices discussed in this report may also be used for the reverse transduction whereby an incoming electromagnetic radiation is rectified to produce an electrical current flowing in the circuitry [23,26]. The interfacing of electron-fed optical antenna with an optical rectenna may thus open a new era for on-chip communication between distant nanometer-scale emitters and receivers [71].
